# Hepatoprotective activity of aerial parts of *Otostegia persica* against carbon tetrachloride-induced liver damage in rats

**Published:** 2015

**Authors:** Mehdi Akbartabar Toori, Behzad Joodi, Heibatollah Sadeghi, Hossein Sadeghi, Mehrzad Jafari, Mohammad Sharif Talebianpoor, Foad Mehraban, Mostafa Mostafazadeh, Mehdi Ghavamizadeh

**Affiliations:** 1 *Social Determinants of Health Research Center, Yasuj University of Medical Sciences, Yasuj, Iran*; 2 *Student Research Committee, Yasuj University of Medical Sciences, Yasuj, Iran *; 3 *Cellular and Molecular Research Center, Faculty of Medicine, Yasuj University of Medical Sciences, Yasuj, Iran*; 4 *Department ofPharmacology,Faculty of Medicine, Yasuj University of Medical Sciences, Yasuj, Iran*; 5 *Cellular and Molecular Research Center, Faculty of Medicine, Yasuj University of Medical Sciences, Yasuj, Iran*

**Keywords:** *Hepatoprotective effect*, *Otostegia persica*, *Liver injury*, *Carbon Tetrachloride*, *Antioxidant property*

## Abstract

**Objective:**

To evaluate the hepatoprotective properties of *Otostegia persica* (*O. persica*) ethanol extract on carbon tetrachloride-induced liver damage in rats.

**Materials and Methods:**

Fifty adult male Wistar rats were randomly divided into five groups. Group I served as normal control and was given only olive oil intraperitoneally (i.p.). Group II, III, IV, and V were administered CCl_4_ mixed with olive oil 1:1 (1 ml/kg) i.p., twice a week for 8 weeks. Group II was maintained as CCl_4_-intoxicated control (hepatotoxic group). Group III, IV, and V received *O. persica* extract at a dose of 40, 80, and 120 mg/kg for 8 weeks every 48 h orally, respectively. Biochemical parameters including aspartate transaminase (AST), alanine transaminase (ALT), alkaline phosphatase (ALP), total bilirubin (TB), albumin (ALB), total protein (TP), and lipid peroxidation marker (Malonaldialdehyde, (MDA) were determined in serum. After 8 weeks, animals were sacrificed, livers dissected out, and evaluated for histomorphological changes.

**Results:**

The administration of CCl4 increased AST, ALT, ALP, TB, and MDA in serum but it decreased TP , and ALB compared with normal control. Treatment with *O. persica* extract at three doses resulted in decreased enzyme markers, bilirubin levels, and lipid peroxidation marker (MDA) and increased TP and ALB compared with CCl_4_ group. The results of pathological study also support the hepatoprotective effects which were observed at doses of 80 and 120 mg/kg.

**Conclusion:**

The results of the present study indicate that ethanol extract of *O. persica* may have hepatoprotective effect which is probably due to its antioxidant property.

## Introduction

Liver diseases are serious health problems. The effectiveness of treatments such as interferon, colchicine, penicillamine, and corticosteroids are inconsistent at best and the incidence of side-effects profound. In the absence of reliable liver protective drugs in allopathic medical practices, herbs play an important role in the management of various liver disorders. A number of plants have shown hepatoprotective property (Luper, 1998 ▶). 

In spite of the tremendous advances in modern medicine, current therapies for hepatic diseases are not very effective yet. Because of the limited therapeutic effects and serious complications of the current medicines for the hepatic diseases, there are a growing focus on exploring novel and alternative approaches for the treatment of liver diseases. Hence a number of medicinal plants and their formulations are used to cure hepatic disorders in traditional medicine (Wagner et al., 1974 ▶; Zhang et al., 2014 ▶). To study the hepatoprotective effects of medicines and plant extracts, CCl_4_-induced hepatotoxicity model is used frequently. The model induces severe liver damage same as viral hepatitis (Rubinstein, 1962 ▶; Sadeghi et al., 2008 ▶). 

The family of Lamiaceae, known as the mint family is one of the most diverse and widespread plant families with about 220 genera and more than 4000 species. This family is important for its flavor, fragrance or medicinal properties (Hedge, 1986 ▶; Naghibi et al., 2005 ▶ ). Some species of the family have been studied for their biological properties. Some studies have determined a variety of valuable activities such as antioxidant and free radical scavenging capacity. 

The genus of *Otostegia* which is a member of this family consists of about 33 species that grows mainly in the Mediterranean regions. Several species of this family are used in traditional and modern medicine. *Otostegia aucheri*, *Otostegia michauxi*, and *Otostegia persica* (*O. persica*) are the three species of the *Otostegia* genus which are endemic in Iran. The *O. persica* or Goldar, locally called "Gol-e-kharu", grows in south of Iran such as Fars, Sistan and Baluchestan, and Kerman provinces (Ayatollahi, 2009 ▶; Sadeghi et al., 2014 ▶; Shrififar et al., 2003 ▶). People of these regions usually use the flower and the aerial parts of the *O. persica* as a food additive and as a traditional medicine to treat inflammatory and rheumatic diseases. 

It is also used as an antihistamin, antispasmotic, antiarthritis, antidiabetic, and antihyperlipidemic agent (Ghahraman, 1996 ▶; Yassa et al., 2005 ▶).

Furthermore, it has been shown that hydroalcoholic extract of *O. persica* improves morphine withdrawal syndrome (Hajhashemi et al., 2004 ▶). Studies have also demonstrated that organic extracts of *O. persica* have antimicrobial effects against Gram-positive bacteria (Asghari et al., 2006 ▶; Tofighi et al., 2009 ▶). Phytochemical studies have shown that there are many chemical components such as flavonoids, steroids, tannins, triterpenoids, and important mineral elements in aerial parts of *O. persica*. Many of these chemical constituents such as flavonoids and related compounds exhibited strong antioxidant activity (Ayatollahi et al., 2007 ▶; Tofighi et al., 2009 ▶).

The extract of aerial part and root of *O. persica* has shown significant hypoglycemic effect in diabetic rats by improving the number of β–cells of pancreatic islets and increasing insulin secretion (Ebrahimpour et al., 2009 ▶; Hedayati et al., 2010 ▶; Hedayati et al., 2011 ▶). In addition, it has been shown that the methanolic shoot extract of *O. persica *has hepatoprotective activity and also can decrease the hepatic dysfunction originated from diabetic disorders (Hedayati et al., 2012 ▶).

According to these properties and folk medicine usage, the aim of the current study was to evaluate the hepatoprotective activity of hydro–alcoholic extract of *O. persica* against CCl_4_-induced hepatotoxicity in experimental rats.

## Materials and Methods

**Chemicals**

CCl_4_ was obtained from Merck and assay kits for the estimation of biochemical factor were purchased from Pars Azema Company, Iran. 

**Animals**

Male Wistar rats weighing 150-250 g were obtained from the animal breeding laboratories of Pasteur Institute (Tehran, Iran). The animals were kept under standard conditions. Housing room was maintained under constant temperature conditions (22±2 ºC), relative humidity (50–60%), and lighting (12-hlight/dark cycle). Food and water were accessible ad libitum (sadeghi et al., 2008). All of the experiments were performed in accordance with the Regulations of Experimental Animal Administration issued by the Ethical Committee of Yasuj University of Medical Sciences (Iran).

**Preparation of extracts**

The aerial parts of the *O. persica *were collected from Firozabad, Fars, Iran. A voucher specimen was deposited in the herbarium of Kohkyloyeh and Boyerahmad Agricultural Research Center. The *O. persica* was powdered in an electrical grinder. The extraction was carried out through the maceration of dry plant powder in ethanol 70% for 48 h at incubation temperature. Then, it was extracted and ethanol was evaporated by rotary evaporator at 40-50 ºC.

The extract was prepared in distilled water before use (Sadeghi and Yazdanparast, 2003 ▶; sadeghi et al. 2008 ▶). Acute oral toxicity study was performed as per OECD guidelines for the testing of chemicals, Test No. 423. Appropriate doses were selected for the present study (OECD, 2001).

**Experimental design**

Fifty adult male Wistar rats were randomly divided into five groups. Group I served as normal control and was given only olive oil i**.**p**., **Group II, III, IV, and V were administered CCl_4_ mixed with olive oil 1:1 (1 ml/kg) i.p., twice a week for 8 weeks. Group II was maintained as CCl_4_-intoxicated control. Groups III, IV, and V were given *O. persica* extract at doses of 40, 80, and 120 mg/kg body weight, respectively. Eight weeks after CCl_4_ administration, and 48 h after last treatment with CCl_4_ and *O. persica* extract, blood was collected by cardiac puncture from all of the animals for biochemical analysis (sadeghi et al., 2008 ▶; Ulican et al., 2003 ▶;).

**Biochemical analysis**

Serum was prepared from the collected blood and subjected to biochemical measurement of different parameters, i.e., AST, ALT, ALP, TB, DB,TP, and ALB (Ulican et al., 2003 ▶; Aniya et al., 2005 ▶) by standard automated techniques using BT 1000 Autoanalyzer and the adequate kits from pars azema, Iran .

In addition, Malonaldialdehyde (MDA) as a lipid peroxidation parameter was measured in serum based on the reaction of thiobarbituric acid with MDA. MDA concentration was determined by comparison to a standard curve of 1,1,3,3-TEP (tetraethoxypropane). Standard curve was made using serial dilution of TEP to yield the following test concentrations: 0, 1, 2, 2.5, 5, and 10 *μ*M. 0.5 mL of serum or standard solutions was taken in a test tube and 2 mL of the TBA (Thiobarbituric acid)–TCA (trichloroacetic acid) (TBA-TCA reagent: 0.375% w/v TBA, 15% w/v TCA, and 0.25 N HCl) solution were added. The mixture was heated in a water bath (90-100 ºC) for 15 min, cooled in a cold water bath for 10 min, and then centrifuged at 2000 g for 15 min. The absorbance of solution was read spectrophotometrically at 535 nm (Hoyland and Taylor, 1991 ▶).

**Histopathological examination**

For the histopathological study, the livers of rats were immediately removed and the tissues were fixed in 10% formalin for a period of at least 24 h. The paraffin sections were then prepared (Automatic Tissue Processor, Shandon, Citadel 1000) and cut into 5-μm thick sections in a rotary microtome. Thereafter, the sections were stained with H&E (haematoxylin and eosin) dye (sadeghi et al. 2008 ▶). The histopathological slides were examined and photographs were taken with a photomicroscope (Olympus Ix71). 

**Statistical analysis**

The results are expressed as mean±SD. The difference between experimental groups was compared using one way ANOVA (Analysis of variance) followed by Tukey’s Post Hoc test using SPSS software version17.

## Results

**Effects of extracts on serum biochemical parameters**

The effects of the *O. persica* extract on biochemical parameters of rats intoxicated by CCl_4_ were evaluated in this study. CCl_4_ was found to cause increases in plasma AST (136.8%) , ALT (192.18%), ALP (89.96%), TB (350%), and MDA (51.14%) levels compared with those in the normal control group (Tables 1 and 2). The serum MDA (48.6%), AST (54.33%), ALT (55.98%), ALP (44.84%), and TB (71.93%) levels were significantly reduced in rats that received the extract of the *O. persica* compared with CCl_4_ group in a dose-dependent manner. However, TP (62.5%) and ALB (79.8%) levels were significantly increased in rats that received the *O. persica* extract with CCl_4_ group (Table 2).

**Table 1:** Effect of hydro alcoholic extract of aerial parts of *O. persica* on various serum biochemical parameters in rats with CCl_4_ induced hepatotoxicity

**Group**	**AST(IU/L)**	**ALT(IU/L)**	**ALP(IU/L)**	**TB (mg/dL)**
**Group I (control)**	183.1±18.7	79.3 ± 8.4	440 ±24.5	0.45 ± 0.01
**Group II (CCl**_4_ **) = hepatotoxic**	433.6±23.2	231.7±27.2	834.3±13.7	2.03±0.05
**Group III (CCl**_4_ **+40 mg/kg ** ***O. persica*** **)**	409.5±38.4	223.4±35.6	528.2±29†	1.17 ±0.07†
**Group IV (CCl**_4_ **+80 mg/kg ** ***O. persica*** **)**	205.9±33.8†	124.1±25.4†	490.3±26†	0.61 ±0.03†
**Group V (CCl**_4_ **+120 mg/kg ** ***O. persica*** **)**	198.5±26†	102 ± 19.9†	460.2±15.1†	0.57 ±0.04†

Values are expressed as mean±S.D. for 10 rats in each group.

† Significant reduction compared to hepatotoxic group (p<0.05).

**Table 2:** Effect of hydro alcoholic extract of aerial parts of O. persica on serum TP, ALB and MDA levels against CCl4 intoxication in rats

**Group**	**TP(g/dL)**	**ALB(g/dL)**	**MDA(nmol/ml)**
**Group I (control) **	7.74±0.18	3.55±0.11	3.05 ± 0.08
**Group II (CCl**_4_ **) = Hepatotoxic**	5.12±0.27	2.13±0.09	4.61±0.27
**Group III (CCl**_4_ **+40 mg/kg ** ***O. persica*** **)**	6.97±0.21*	3.31±0.15*	3.19 ±0.22†
**Group IV (CCl**_4_ **+80 mg/kg ** ***O. persica*** **)**	8.18±0.27*	3.65±0.09*	2.84 ±0.15†
**Group V (CCl**_4_ **+120 mg/kg ** ***O. persica*** **)**	8.32 ±0.15*	3.83 ±0.12*	2.37 ±0.14†

Values are expressed as mean±S.D. for 10 rats in each group.

* Significant increase compared to hepatotoxic group (p<0.05).

† Significant reduction compared to hepatotoxic group (p<0.05).

**Figure 1:**
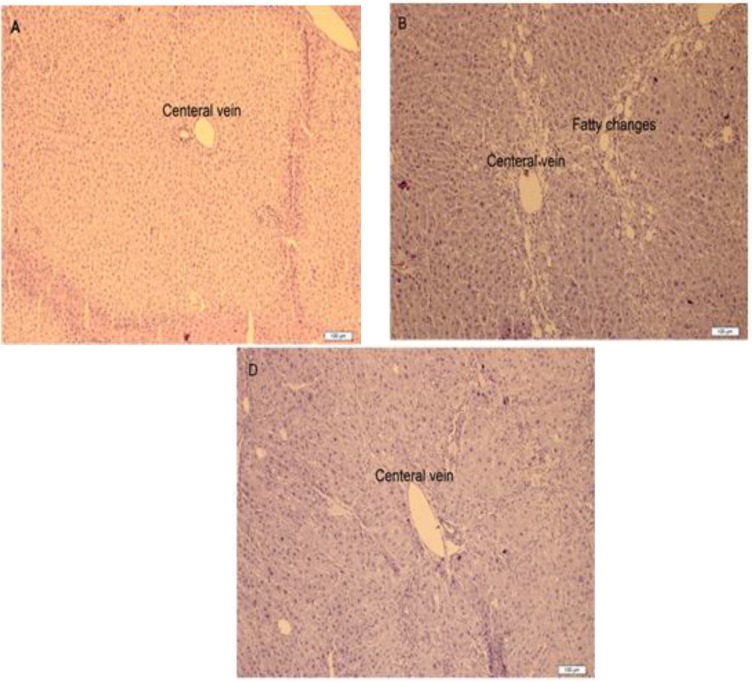
Liver histopathology of rats treated with CCl_4_ and *O. persica* extract. The liver section of each rat from different groups was stained by haematoxylin and eosin (H&E) staining, and the images were examined under light microscope. The photographs show the pathological changes in hepatic tissues (original magnification of 100): (A) Normal group, (B) CCl_4_+ olive oil group, and (D) 80 mg/kg B.W. of *O. persica* extract + CCl_4_ group.

**Histopathological examination of the liver**

As shown in Figure 1, the hepatic tissues in rats of the control group exhibited normal cellular structure with distinct hepatic cells and sinusoidal spaces structure. In contrast, the liver of CCl_4_-treated group exhibited the most severe damage of all groups, where the liver sections in this group showed congestion with sinusoids dilatation and ballooning degeneration (especially at the periphery of lobules). However, the pathological hepatic lesions induced by the administration of CCl_4_ were remarkably ameliorated by *O. persica* extract in a dose-dependent manner and this was in good agreement with the results of serum biochemical parameters and hepatic oxidative stress level. The maximum protection was observed at the dose of 80 mg/kg B.W. of *O. persica* extract and the liver sections of the rats from these groups showed minor patho-morphological changes that were more similar to the control group.

## Discussion

CCl_4_ is one of the most powerful hepatotoxins which is able to induce liver damage through the formation of reactive free radicals such as trichloromethyl (CCl_3_) or trichloroperoxyl radicals (CCl_3_ O°_3_). It can cause severe damages to the liver such as fatty changes centrilobular steatosis, inflammation, apoptosis, and cell necrosis (Lutz et al., 2003 ▶; Sadeghi et al., 2008 ▶ ). Therefore, the main intercellular structures which are affected by CCl_4_ are plasma membrane, endoplasmic reticulum, mitochondria, and Golgi apparatus. (Reynolds, 1963 ▶). As a result of damaging the cell membrane of hepatocytes, enzymes release in circulation (Cullen, 2005 ▶). In the CCl_4_-treated group, the levels of ALT, AST, ALP, TB, and MDA increased and the levels of TP and ALB reduced compared to the normal control group, indicating severe hepatocellular damage (Tables 1 and 2). 

The signs of hepatoprotective effects of a biological agent are to maintain the normal physiological function of hepatocytes and reduce the damage of intercellular structures from exposure to the toxic agent (Balderas et al., 2007 ▶; Hui et al., 2008 ▶; Omolola and Ebenezer, 2010 ▶).

Administration of *O. persica* ethanol extract at concentrations of 80 and 120 mg/kg, for eight weeks resulted in significant (p<0.05) reduction of CCl_4_-induced elevation of serum enzyme markers (Tables 1 and 2), comparable to the effect of several plants that have been examined for use in a wide variety of liver disorders such as *Silybum marianum*, *Picrorhiza kurroa*, *Curcuma longa*, *Camellia sinensis Chelidonium majus, *and *Allium sativa* (Balderas et al., 2007 ▶; Hui et al., 2008 ▶; Omolola and Ebenezer, 2010 ▶; Luper, 1998 ▶ ). 

Through the action of cytochrome P_450_ oxygenase system, CCl_4_ metabolism begins with formation of tricholoro methyl free radical CCl_3_^•^. Both CYP2E1 and CYP3A are cytochrome P_450_ isoenzymes that contribute significantly in this activation (McCay, 1984 ▶). In the presence of oxygen, CCl_3_^•^ radical is converted to trichlromethyl peroxy radical CCl_3_COO^•^ which is more active than CCl_3_^•^. Both radicals can react with different substances such as proteins, nucleic acids, and lipids and damaged their normal functions. 

They can start the process of lipid peroxidation by reducing hydrogen from polyunsaturated fatty acids. This process by compromising membrane function and covalent binding of reactive intermediate can lead to liver cell necrosis (Recknagel, 1983 ▶; Recknagel et al., 1989 ▶). Studies have shown that CCl_4_ can increase the levels of Ca^2+^ in cells. This increase can activate many catabolic enzymes that destroy cytoskeletal construction and cell death through apoptosis or necrosis (Houzi et al., 2000 ▶; Nicotera et al., 1992 ▶). 

MDA is an end product of lipid peroxidation which is known as a marker of oxidative stress (Pramod et al., 2008 ▶; Neetu and Sangeeta, 2011 ▶). In this study CCl_4_ increased MDA level in group II which is in agreement with other studies and treatment with *O. persica* extract decreased the levels of MDA in groups III to V. These results show that *O. persica* extract can ameliorate the oxidative stress induced by CCl_4_. 

Five compounds were isolated and purified from the methanol extract of *O. persica*. Four of them, kampferol, morin, quercetin, and isovitexin, were identified as flavonoids. These flavonoids and related compounds showed significant antioxidant activities that are comparable to butylated hydroxyanisole (BHA) and vitamin E (Shrififar et al., 2003 ▶). Antioxidants are compounds that protect organism against oxidative stress by scavenging free radicals compounds and inhibit the oxidative mechanisms that lead to degenerative diseases such as atherosclerosis, liver disease, diabetes, aging, and cancer (Sheweita et al., 2001 ▶).

Morin is a kind of flavonoid found in the plants of Moraceae family which are used as dietary agents in herbal medicine (Sreedharan et al., 2009 ▶). It has various biological effects including antioxidant properties, xanthine oxidase and protein kinase C inhibitor, anticancer, and anti-inflammatory effects (Subash and Subramanian, 2009 ▶). Moreover, morin acts as an inhibitor of acute liver damage by blocking the expressions of inflammatory cytokines and mediators including TNF-α, IL-1β, IL-6, and iNOS (Lee et al., 2008 ▶). In addition, quercetin is one of the most abundant flavonoid compounds that distributed as secondary metabolites in many plants (Liu et al., 2012 ▶). It has also shown to possess anti-inflammatory, anti-allergic, anti-cancer, cardioprotective, and potent antioxidant activities (Leopoldini et al., 2006 ▶). The anti-inflammatory activities of quercetin may arise from its inhibitory effects on cyclooxygenase (COX) and lipo-oxygenase (LOX) enzymes (Joshi et al., 2011 ▶). Quercetin is considered to be a strong antioxidant due to scavenging free radicals and chelating transition metal ions that inhibits lipid peroxidation and free radical production (Coşkun et al., 2004 ▶).

The results of histopathological study also support the results of hepatoprotective effect of *O. persica* extract which were observed at doses of 80 and 120 mg/kg. Simultaneous treatment of *O. persica* extract with CCl_4_ showed significantly less damage to the hepatic cells compared to rats treated with CCl_4_ alone. The reduction in cellular damage seen in *O. persica* extract-treated group was morphologically similar to the control group (Figure 1). Hepatoprotective mechanisms of *O. persica* on CCl_4_-induced acute liver damage might be due to the decreased lipid peroxidation (Nasiri Bezenjani et al., 2012 ▶).

In conclusion, the results of this study demonstrated that *O. persica* extract is effective for the prevention of CCl_4_-induced hepatic damage in rats and therefore it could be used as a hepatoprotective agent. The protective effects against liver damage may be, at least in part, due to the free radical scavenging effect, inhibition of lipid peroxidation, and increased antioxidant activity (Yassa et al., 2005 ▶). Further studies with individual active compounds of *O. persica* are needed to understand the exact mechanism of hepatoprotective action.
